# How Green Transformational Leadership Affects Employee Individual Green Performance—A Multilevel Moderated Mediation Model

**DOI:** 10.3390/bs13110887

**Published:** 2023-10-27

**Authors:** Haoming Ding, Wei Su, Juhee Hahn

**Affiliations:** 1 The Graduate School, Hoseo University, Asan 31499, Republic of Korea; dinghaoming123@naver.com; 2 The Graduate School, Chung-Ang University, Seoul 06974, Republic of Korea; suv4591@gmail.com; 3 Department of Business Management, Chung-Ang University, Seoul 06974, Republic of Korea

**Keywords:** green transformational leadership, green creativity, creative process engagement, individual green performance, individual environmental awareness

## Abstract

Rapid economic growth puts the natural environment under tremendous pressure. As a traditional chemical company, it is important to reconsider outdated business development models, develop innovative green initiatives for long-term growth, and choose approaches to address environmental issues. Determining how to encourage employees’ green performance while balancing environmental issues is crucial for chemical companies in the current social and economic environment. This study investigates the green transformational leadership style to enhance green performance of chemical company employees. It expands the field of environmental protection by employing two novel constructs: creative process engagement and green creativity. We collected 623 valid questionnaires from 98 teams (98 leaders and 525 employees) and used SPSS 26.0, HLM 6.0, and MPlus 8.3 to test the hypothesis. The findings revealed that (1) green transformational leadership positively influences individual green performance, (2) creative process engagement and green creativity mediate the relationship between green transformational leadership and individual green performance, and (3) individual environmental awareness positively moderates the relationship between green transformational leadership and green creativity. These novel findings contribute to the environmental literature and help chemical company managers in enhancing employee innovation and performance.

## 1. Introduction

Increasing environmental pressure and natural deterioration have emerged as formidable obstacles for humanity. Sustainable development and environmental issues are becoming increasingly important to the international community, academia, and industry [1]. Chemical companies pollute the environment more than other industries, and we urgently need a new leadership management model to lead chemical companies to change the environmental field, and reform and innovate their management models [2]. This study aims to examine how GTL affects employees’ individual green performance in the context of environmental protection in a Chinese chemical company.

Green transformational leaders’ pro-environmental behaviors in the workplace can subtly shape employees’ cognitions to gradually comprehend and learn the environmental protection values conveyed by leaders. Through the leaders’ environmental consciousness, green transformational leadership manifests in chemical enterprises. In addition, it communicates the company’s environmental vision to employees and serves as a role model for explaining corporate environmental values and taking appropriate measures to solve environmental problems [3]. Therefore, green transformational leaders in chemical companies can change their environmental field, improve their employees’ green performance, and inject new impetus into corporate reform and innovation. GTL, IGP and environmental sustainability efforts can be better understood through the lens of social exchange theory (SET) [4]. Social exchange theory and the concept of norms of reciprocity have been utilized for many years to understand employee attitudes and behaviors. Exchange was created based on the principle of reciprocity emphasized by social exchange theory. Leader–member exchange is the key to social exchange relationships in organizations. Transformational leaders strengthen the quality of reciprocal relationships with their followers by demonstrating care, trust, and support. When employees perceive that the leader provides valuable resources and support, a sense of reciprocity develops. This care emanates from the transformational leader and leads to a high-quality reciprocal relationship between the employee and the leader [5]. Therefore, according to social exchange theory, GTL can effectively motivate employees to demonstrate more IGP behaviors by articulating a vision that boosts their confidence and expectations.

The environmental performance of an organization’s employees over a predetermined amount of time is known as “green performance”. Adopting green practices increases chemical companies’ long-term competitiveness and reduces the cost of energy consumption (such as electricity and water costs) to achieve sustainable development [6]. The green performance of each employee is critical to ensuring business compliance with regulations and standards [7]. In addition to developing chemical enterprises, enhancing employees’ green performance in chemical enterprises is an urgent issue that must be resolved. 

Employees’ green creativity is a prerequisite for green performance, affects enterprises, and is crucial to their ability to gain a competitive advantage. Therefore, based on previous findings on creativity, scholars focus on improving employees’ green creativity [8]. In environmental management research, the starting point for green organizational innovation is employees’ green performance. It can help organizations adapt to a changing economic environment while maintaining high motivation for green innovation performance [9]. The performance of green creativity in chemical companies is usually reflected in developing original, innovative, and practical new ideas related to green products, green services, green production processes, or green practices [6]. Therefore, the essential issues for study and resolution are whether the leadership of chemical companies can effectively identify, motivate, and cultivate the green creativity of employees, and the advantages of employees with green performance over ordinary employees [10]. Introducing green creativity as the first mediator variable in this investigation of the impact of green transformational leadership on individual green performance creates a new precedent. 

Creative process engagement refers to the active participation of employees in the innovation approach and process, and the demonstration of activity in three basic dimensions: problem identification (employees are able to identify problems or challenges that exist and are aware of the significance of solving them [11]); information processing (employees must comprehend the nature and context of the problem to be able to think critically and generate ideas [12]); and solution generation (based on the understanding of the problem, employees can generate new ideas using creativity to solve it [13]). When working in chemical enterprises where employees invest more heavily in creativity-related methods and processes, it is advantageous for employees to create fresh concepts about environmental protection awareness and encourage organizational members to engage in these creative green behaviors [14]. To further investigate the pathways and moderators of the effect of green transformational leadership on individual green performance, we can include creative process engagement in this variable to deepen our understanding of this relationship. Therefore, the second mediating variable in this study of the impact of green transformational leadership on individual green performance is creative process engagement. 

Through their actions, leaders can influence their teams’ environmental performance. However, environmentally conscious leaders will encourage their teams’ environmental impact and participation in various environmental protection activities. Employees are environmentally aware when they understand environmental issues, can change their behavior, and recognize environmental awareness and its causes [15]. These factors speak to a person’s understanding of environmental issues and potential solutions. At work, individuals who care about the environment act in environmentally friendly ways. According to studies, employees are more likely to engage in green behaviors at work when they are aware of ecological and environmental issues [16]. 

Individual environmental awareness is a crucial element of transformational leadership and employee green creativity. Individual environmental awareness is vital for perception, participation, compliance, innovation, improvement, employee engagement and satisfaction, and corporate image for chemical companies. Further research is required to validate and investigate in-depth the specific moderating mechanisms and effects of individual environmental awareness on the relationship between green leadership and green creativity. As a result, the research model for this thesis included individual environmental awareness as a moderating variable for green transformational leadership and green creativity. 

Compared to previous studies, the need for chemical companies to improve the environment is more urgent, and this study can provide an important reference for chemical companies to help them to transform, improve environmental protection, and fill the research gap relating to chemical companies. Chemical company leaders should focus on green transformational leadership to influence the environmental behavior of their employees and encourage them to achieve individual green performance. Following this logic, this study investigated the effect of green transformational leadership on the individual green performance of Chinese chemical company employees. In addition, this study focuses on examining the chain-mediating role of green transformational leadership on individual green performance between creative process engagement and green creativity, as it has received less attention in previous studies. For this reason, we fill the research gap by constructing a novel research framework and provide a series of relevant insights for Chinese chemical companies.

In summary, the following are the key questions of this study:

Q1. What role does GTL play in enhancing employees’ individual green performance?

Q2. What role do green creativity and creative process engagement play in the impact of GTL practices on individual employees’ green performance?

Q3. Does individual environmental awareness as a personal characteristic amplify or weaken the relationship between GTL and green creativity?

To achieve the research objectives and answer the research questions, the article is structured as follows. In Section 2, we provide the theoretical framework of the study. In Section 3, we describe the research methodology, including the procedures we used to collect and examine the research data. In Section 4, we describe the study’s conclusions. Section 5 summarizes the conclusions and the managerial and theoretical implications of the study, points out its limitations, and makes recommendations for future research.

## 2. Theoretical Foundation and Hypotheses Development 

### 2.1. Green Transformational Leadership and Individual Green Performance 

Leaders play a role in the success of environmental initiatives [17]. A growing number of researchers have realized the value of leadership for personal and environmental performance [18]. We can observe the positive effects of green transformational leadership on green performance by implementing employee motivational elements such as inspirational motivation and intellectual stimulation [19]. Transformational leaders encourage their followers to make breakthroughs by developing novel ideas. According to studies, transformational leadership encourages employees to invent new problem-solving strategies and facilitates the generation of new ideas [20]. Transformational leadership enables team members to think about challenges from several perspectives by engaging in green behaviors within a set timeframe. 

Specifically, green transformational leaders can highlight areas where the company needs to improve in environmental protection and present a better future vision to employees to gain their respect and boost their confidence in their ability to contribute to green transformation and innovation. They can also propose breaking the rules, pique employees’ curiosity, and motivate them to be creative [17]. Green transformational leaders also set an example for others by promoting innovation at work, modeling innovative behavior, and directing and motivating staff to express their green creativity. Leaders who inspire confidence in their employees have employees who tend to take more risks in green creation and innovation. 

Moreover, prior studies have referred to the performance of a company’s hardware and software used in green product or process innovations as “green innovation performance” [19]. It comprises technological innovation, energy conservation, waste recycling, pollution prevention and control, green product design, and corporate environmental management among chemical companies [9]. These findings suggest that green transformational leadership can positively influence individual green performance. Thus, by shaping organizational culture, inspiring environmental responsibility and commitment among employees, and providing support and training, this leadership style can lead to more active engagement in environmentally friendly behaviors, thereby enhancing individual green performance. Based on the above, we assert that green transformational leadership has a positive effect on green performance. Based on prior research, this study suggests the following hypothesis:

**Hypothesis 1.**  *Green transformational leadership will positively impact individual green performance.*

### 2.2. The Mediating Role of Creative Process Engagement 

Creative process engagement is primarily a cognitive, behavioral, and productive state in which people recognize problems, seek information and develop solutions [21]. People engage in the creative process when they brainstorm, discuss ideas, and collaborate. Although time consuming, this process needs a setting that encourages taking chances and attempting novel solutions to longstanding issues. Through their vision, knowledge, individualized mentoring, supportive culture, and capacity for intellectual stimulation, transformational leaders can persuade their team members to engage in creative endeavors, fostering creative impact [6]. Green transformational leadership positively impacts employee participatory innovation behavior [20]. This leadership style promotes active participation and contributions by encouraging employees to express their opinions, participate in decision making, and share ideas to drive innovation activities [22]. By creating a supportive environment for innovative and creative behavior, leaders can motivate their employees to participate in the creative process [23,24]. 

The existing literature presents findings regarding the relationship between green transformational leadership and creative process engagement. Previous research has demonstrated that visionary leadership in green transformational leadership supports and fosters a workplace environment that makes it easier to identify issues and develop creative solutions [24]. These findings support the positive impact of green transformational leadership on creative process engagement [25]. Green transformational leadership promotes the development of creative process engagement by encouraging innovative thinking, providing support and resources, and promoting environmental values that stimulate employee creativity, participatory innovation behaviors, and team creativity [26,27]. 

To generate original and valuable ideas, team members must devote considerable time and effort to problem identification, information gathering and processing, solution formation, selection, implementation, and encouraging a higher level of team reflection [28]. Green transformational leadership can influence and support the definition of issues, information seeking, and idea generation [29]. Based on the innovation process perspective, creative problem-solving requires extensive and in-depth cognitive processing [30]. Individuals or groups must identify and precisely define the problem, actively collect information related to the problem, and encode and interpret the information. According to Zhang et al. (2022), creative thinking can help individuals tasked with solving a problem in clarifying the issue and gathering relevant information. From there, the creative process can help them to generate and evaluate potential solutions to identify the best strategy. 

Brainstorming facilitates the generation of new ideas and putting them into practice, which improves the creative performance of individuals or groups [31]. Team members expend time and effort to define the problem and acquire pertinent information to solve the problem, which may result in more novel and practical ideas [32]. Applying positive emotional and cognitive resources to innovative thinking activities can stimulate team members’ creative potential, help members to comprehend the problem substantively, collect and analyze pertinent data exhaustively, and enhance the originality of problem solutions [33]. Creative process engagement is a prerequisite for innovation. Individuals and groups need to invest time and effort continuously from the generation of ideas to their perfection [34]. The greater the level of creative process investment, the greater the possibility of the team generating novel ideas and the higher the employees’ green performance [35]. Therefore, the following hypotheses are presented in this study:

**Hypothesis 2.**  *Translating green leadership will positively impact creative process engagement.*

**Hypothesis 3.**  *Creative process engagement will mediate the relationship between green transformational leadership and individual green performance.*

### 2.3. The Mediating Role of Green Creativity 

Green transformational leadership entails encouraging employees to go above and beyond expected environmental performance. For example, transformational leadership in environmental protection is green transformational leadership [36]. Environmental influence refers to leaders setting an example, engaging in environmentally friendly behaviors, demonstrating their dedication to environmental protection through concrete actions, and serving as role models for their employees. In addition, green transformational leaders create environmental vision incentives for employees by communicating sustainable development to motivate employees [6]. Therefore, green transformational leadership can encourage the realization of green environmental protection goals, inspire organizational members to engage in green creation, and raise the company’s green performance to a level above expectations [37]. 

Green transformational leaders also exhibit environmental protection in their professional activities. They do this by being creative, setting an example for others, and directing and motivating staff to expand their green creativity [38]. A positive association exists between green transformational leaders and employee green innovation behaviors. For instance, green transformation leaders can drive positive employee behavior in green innovation by providing environmental support, encouraging creative thinking, and advocating green innovation goals [39]. Furthermore, by supporting and encouraging employee green innovation behaviors and providing resources and environmental support, green transformational leaders can inspire green idea generation and drive organizational innovation and improvement in sustainability [40]. 

According to earlier studies, green creativity can improve a company’s environmental performance and is a significant factor in determining the success of innovative projects within an organization [41]. Green creativity can help staff members develop original and helpful ideas—thus, employees may benefit from an organizational concept that encourages green creativity [42]. As a result, green creativity plays a crucial role in driving green performance [43]. Therefore, the team’s green innovation capability mediates the relationship between green transformational leadership and individual green performance. Green transformational leaders indirectly influence individual green performance through the team’s green innovation capacity [39]. 

By fostering a work environment that motivates employees to perform better, leaders in chemical companies can exercise effective green transformational leadership and foster employees’ green creativity [44]. Employees who feel trusted and have confidence in their leaders are more willing to take on the risks involved in green innovation and creation [45]. Green transformational leadership encourages employees to learn new skills and technologies and drives them to adopt innovative green practices, enabling the company to sell environmentally friendly goods [46]. Additionally, green transformational leadership persuades staff members to put corporate green goals ahead of personal goals. It provides them with all the tools they need to generate fresh suggestions for environmental improvement [46]. Therefore, employees produce new ideas when their leaders encourage and support their innovative ideas and imaginative visions [47]—green transformational leadership is necessary for directing and inspiring staff [48]. Therefore, based on previous research, this study presents the following hypotheses:

**Hypothesis 4.**  *Green transformational leadership will positively impact green creativity.*

**Hypothesis 5.**  *Green creativity will mediate the relationship between green transformational leadership and individual green performance.*

### 2.4. The Sequential Mediation Effects of Creative Process Engagement and Green Creativity 

The research shows that green transformational leadership has a favorable effect on creative process engagement. Numerous studies have identified transformational leadership as a catalyst for creative process engagement [49]. In addition, studies show that green transformational leadership improves environmental outcomes [35]. The resource conservation theory claims that employees develop innovative solutions while contributing and acquiring significant resources. Thus, encouraging employees to participate in green creativity and performance can effectively promote green innovation in enterprises. Therefore, green transformational leadership can encourage employee creativity and simultaneously provide employees with more relaxed working conditions [50]. Prior research has also demonstrated that creative process engagement positively affects green creativity. For instance, Faupel et al. (2012) revealed the positive effects of the creative process on green performance and other areas through green creativity. Through green creativity and creative process engagement, green transformational leadership can impact employees’ green performance in chemical enterprises. 

Although these studies do not directly explore the sequential mediating effects of creative process engagement and green creativity, their findings provide clues to understanding the relationship between green transformational leadership, creative process engagement, green creativity, and individual green performance. Further research may need to explore the mechanisms by which creative process engagement and green creativity play a role in this relationship and further validate and extend these findings. Considering the above presumptions, this study theorizes that creative process engagement and green creativity may mediate between transformational leadership and individual green performance, specifically “green transformational leadership to creative process engagement to green creativity to individual green performance”. Therefore, we offer the following hypothesis:

**Hypothesis 6.**  *Green transformational leadership will positively impact individual green performance through the sequential mediation effects of creative process engagement and green creativity.*

### 2.5. The Moderating Role of Individual Environmental Awareness 

Studies have shown that people’s surroundings may impact their actions in several ways, including knowledge, awareness, and concern for others. Various factors influence whether people contribute directly or indirectly to green creativity [51]. Previous research has shown that the effect of green leadership on creativity is more significant when individual environmental concern is higher [52]. Direct influences on employee green creativity include a sense of obligation and environmental awareness, a sense of mission and belonging, concern for the neighborhood, and a sense of moral responsibility [53]. Employees with high environmental awareness are more likely to adopt environmentally friendly workplace practices because they can weigh the costs and advantages of environmental issues [54]. Employees in a chemical company, for instance, will consciously engage in behaviors related to the environment and reduce activities related to environmental damage when they are more environmentally conscious. 

When people are aware of environmental problems, they are more inclined to take action to prevent them [55]. Environmental pollution causes chemical companies to emphasize the importance of the environment [23]. Due to their awareness of the environment, the information and requirements can be straightforward when green transformational leaders propose new standards and environmental protection measures. However, leaders should quickly communicate the information to encourage staff members’ environmental creativity [21]. Therefore, environmentally aware leaders are more likely to inspire and generate green creativity. Thus, environmental consciousness moderates the relationship between green transformational leadership and green creativity. Accordingly, based on the literature, this study presents the following hypothesis:

**Hypothesis 7.**  *Individual environmental awareness will moderate the relationship between green transformational leadership and green creativity. High individual environmental awareness will strengthen the positive impact of green transformational leadership on green creativity.*

**Figure 1:**
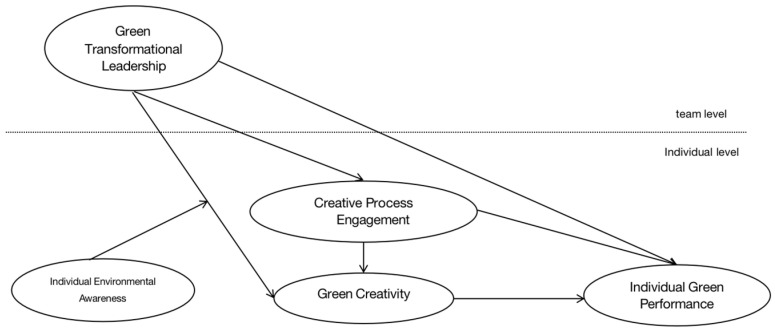
Theoretical Framework.

As shown in Figure 1, this study will examine the effect of green transformational leadership on individual employee green performance and the chain-mediated mechanisms of creative process engagement and green creativity in this main effect. In addition, the boundary effect of individual environmental awareness is added to the relationship between green transformational leadership and employee green creativity.

## 3. Methods

### 3.1. Sample and Procedure

This study is deductive and quantitative, and we have used a convenience sample, which is a non-probability sampling technique. This study’s survey data came from team leaders and employees at five chemical companies in the Shandong province of China. The data collection began on 16 July 2022, and ended on 13 September 2022. In accordance with previous studies, a time-lagged research method was used to reduce common method bias and improve the data quality and credibility of the questionnaire survey [56]. We used time-lagged research methodology to conduct the data survey in four phases to ensure more accurate and reliable results. Each stage had a two-week data collection period. To collect survey data more effectively, we requested the participation of several chemical companies’ presidents and human resources directors by email and telephone. The study received letters from five companies permitting the study’s data collection to proceed as planned. Before participating in the questionnaire, all participants were informed of the purpose of the study, and data confidentiality was ensured. The questionnaire was allowed to be administered with the consent of the participants. As all participants were Chinese, the questionnaire was translated from English to Chinese and then back to English.

The first questionnaire survey in this study was conducted from 16 July 2022 to 28 July 2022, with employees as respondents. This survey allowed employees to evaluate their leaders. Employees received an invitation from their leaders to participate in the green transformational leadership questionnaire survey. In the first survey, 564 completed questionnaires were collected.

The second questionnaire survey was conducted from 29 July 2022 to 13 August 2022, with employees as respondents. The questionnaire contents asked for employee self-evaluations. Like the first survey, the employees’ leaders invited them to participate in the individual environmental awareness and creative process engagement questionnaires. In this round, 551 completed employee questionnaires were collected. 

The third questionnaire survey was conducted from 14 August 2022 to 28 August 2022, with employees as respondents. The questionnaire contents asked for employee self-evaluations. Again, their leaders invited the employees to complete the green creativity questionnaires. In this third survey, a total of 535 completed questionnaires were collected.

The fourth questionnaire survey was conducted from 29 August 2022 to 13 September 2022, with leaders as respondents. The questionnaire asked leaders to evaluate their employees. The companies’ directors invited the leaders to participate in the individual green performance questionnaire survey. In this round, a total of 532 completed leader questionnaires were collected.

Finally, after removing the missing and invalid questionnaires, 98 valid group leader questionnaires, and 525 valid employee questionnaires were obtained. In addition, to verify the unique participant responses that were received, the survey form requested the participants to write the last four digits of their mobile phone numbers and to provide their team numbers. 

Appendix A (Table A1) presents the operationalized questionnaire.

### 3.2. Measures 

#### 3.2.1. Green Transformational Leadership

The green transformational leadership measurement relied on a six-item scale reported by Chen et al. [6]. A sample measurement item is “My leader gets the employees to work together for the same environmental goals.” Participants responded to the questionnaire using a five-point Likert scale, with responses ranging from “1 = strongly disagree” to “5 = strongly agree”. 

#### 3.2.2. Creative Process Engagement 

The creative process engagement measurement used an 11-item scale from Zhang and Bartol [12]. A sample measurement item is “I spend considerable time trying to understand the nature of the problem.” Participants used a five-point Likert scale, with responses ranging from “1 = strongly disagree” to “5 = strongly agree”. 

#### 3.2.3. Green Creativity 

Unlike innovative behavior, green creativity is not a specific, observable behavior. Leaders do not fully observe employees’ green creativity in their daily work; thus, they rely on employee self-evaluations. This study’s green creativity measurement included tools from Mittal and Dhar [35], consisting of six questions. Respondents used a five-point Likert scale, with responses ranging from “1 = strongly disagree” to “5 = strongly agree”. A sample item is “I can come up with new ways to achieve environmental goals.” 

#### 3.2.4. Individual Environmental Awareness 

The individual environmental awareness measurement included a four-item scale reported by Thomson et al. [57]. A sample item is “Environmental protection will provide a better world for me and my children.” Employee participants responded to this questionnaire using a five-point Likert scale, with responses ranging from “1 = strongly disagree” to “5 = strongly agree”. 

#### 3.2.5. Individual Green Performance 

The individual green performance measurement used a ten-item scale from Tuan [58]. A sample measurement item is “In his/her work, this employee weighs the consequences of his/her actions before doing something that could affect the environment.” Team leader participants responded to questions using a five-point Likert scale, with responses ranging from “1 = strongly disagree” to “5 = strongly agree”.

## 4. Data Analysis

### 4.1. Descriptive Results

As shown in Table 1, this study used SPSS 26.0 to conduct descriptive statistics on the essential demographic characteristics of team leaders and employees. 

### 4.2. Preliminary Analyses

Table 2 demonstrates the reliability and validity scores of each variable. The Cronbach’s alpha values for all variables exceeded the 0.70 threshold, indicating internal consistency. Similarly, the AVE values exceeded 0.50, and the CR values were greater than 0.80; consequently, all variables’ reliability and validity scores were sufficient for further study.

Table 3 shows the correlations, means, standard deviations, and square roots of the AVE values for team leader and team member data. The results indicate the suitability of team leader and team member data for further analysis; there were no severe correlation problems, and the square root of the AVE value was higher than the correlation coefficients of the variables.

### 4.3. Hypothesis Tests

We aggregated the individual perceptions of green transformational leadership (estimated cluster averages) to form a team-level measure of team green transformational leadership [22]. The Rwg of team green transformational leadership in this study was 0.736, exceeding the threshold of 0.70, indicating homogeneity of green transformational leadership perception of 73.6%, which is good for within-group consistency. The ICC1 value for green transformational leadership was 0.502, indicating that differences between teams explained 50.2% of the variance in green transformational leadership. The ICC2 value was 0.844 (>0.7), showing that the reliability of the group mean for in-sample green transformational leadership is valid. The results suggest that conceptualizing and analyzing green transformational leadership at the team level is statistically appropriate. 

We conducted null model testing before cross-level analysis to assess whether grouping variables at level 2 significantly impacted level 1’s dependent variable. We could do this by evaluating the intraclass correlation coefficient (ICC) value, showing the proportion of variance in the hierarchical model. Table 3 shows the findings of the null model testing. In null model 1, the team level explained 45.4% (0.276/(0.332 + 0.276) = 0.454) of the variance in employee creative process engagement. As shown in null model 2, the team level explained 50.4% (0.400/(0.393 + 0.400) = 0.504) of the variance in employee green creativity. Moreover, as shown in null model 3, the team level explained 41.1% (0.294/(0.422 + 0.294) = 0.411) of the variance in employee green creativity. We confirmed this result with multilevel confirmatory factor analysis, as follows: CMIN/DF = 1.452 (less than 3), CFI = 0.976 (above 0.9), TLI = 0.974 (above 0.9), RMSEA = 0.029 (less than 0.08), SRMR = 0.034 (less than 0.08). Thus, the reliability and validity of the model fit were acceptable.

Model 7 in Table 4 analyzes the impact of control variables on individual green performance, and the results show that control variables have no significant effect on individual green performance. Model 8 is based on Model 7, with the addition of the independent variable green transformational leadership. The results indicate that the newly added team variable, green transformational leadership, significantly affects the individual variable, individual green performance (γ = 0.540, *p* < 0.001). This finding supports H1. 

Model 1’s results in Table 4 show that the control variables do not impact creative process engagement. Model 2 adds the team-level independent variable green transformational leadership, based on Model 1. The result indicates that green transformational leadership positively affected creative process engagement (γ = 0.379, *p* < 0.001), supporting H2. Based on Model 8, when we added creative process engagement (mediator) into Model 9, the positive relationship between green transformational leadership and employee individual green performance decreased (γ = 0.348, *p* < 0.001). In contrast, creative process engagement and employee individual green performance (γ = 0.504 ***, *p* < 0.001) had a positive relationship. This demonstrated the partial mediation of creative process engagement between green transformational leadership and employee individual green performance, supporting H3. 

Model 3 analyzes the impact of control variables on green creativity, and the results show that control variables do not affect creative process engagement. Model 4 adds the team-level independent variable green transformational leadership, based on model 3. The regression coefficient shows that green transformational leadership positively impacts green creativity (γ = 0.549, *p* < 0.001), supporting H4. 

Based on Model 8, when we added green creativity (mediator) into Model 10, the positive relationship between green transformational leadership and employee individual green performance decreased (γ = 0.258, *p* < 0.001). In contrast, green creativity and employee individual green performance (γ = 0.511 ***, *p* < 0.001) had a positive relationship. It indicated the partial mediation of green creativity between green transformational leadership and employee individual green performance, supporting H5. 

Based on Model 9, we added green creativity into Model 11. The resulting coefficient shows that green creativity positively impacts individual green performance (γ= 0.390, *p* < 0.001). The impact of the independent variable green transformational leadership decreases from 0.540 to 0.348 and further decreases to 0.194, The effect of the first mediating variable, creative process engagement, decreases from 0.504 to 0.344, indicating a sequential mediation effect. This result supports H6. 

In this study, we performed hypothesis testing using a hierarchical interaction method to investigate whether individual environmental awareness moderates the analysis of the effect of green transformational leadership on green creativity. Table 3 shows that Model 5 adds the moderating variable of individual environmental awareness based on Model 4, and the results show that individual environmental awareness positively affects green creativity (γ = 0.237, *p* < 0.001). Model 6 is based on Model 5 by adding interactive green transformational leadership and individual environmental awareness. The results show that the added interaction variable (green transformational leadership × individual environmental awareness) positively affects green creativity (γ = 0.347, *p* < 0.001). Therefore, this result supports H7.

**Figure 2:**
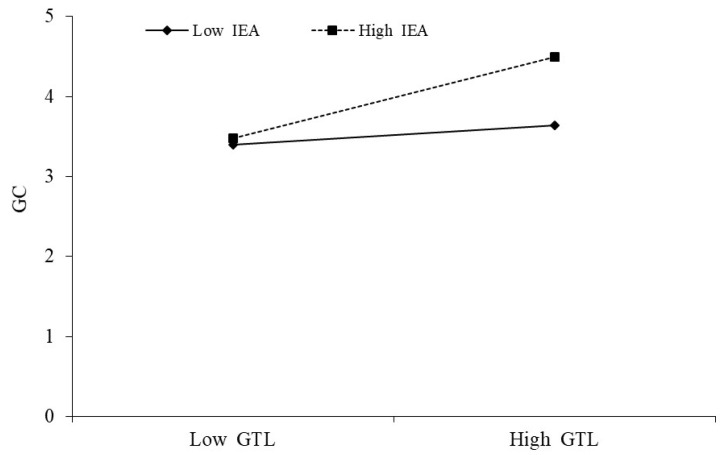
Moderation of individual environmental awareness between green transformational leadership and green creativity relationship.

Figure 2 illustrates the presence of environmental awareness in the relationship between green transformational leadership and green creativity. 

### 4.4. Robustness Tests 

**Figure 3:**
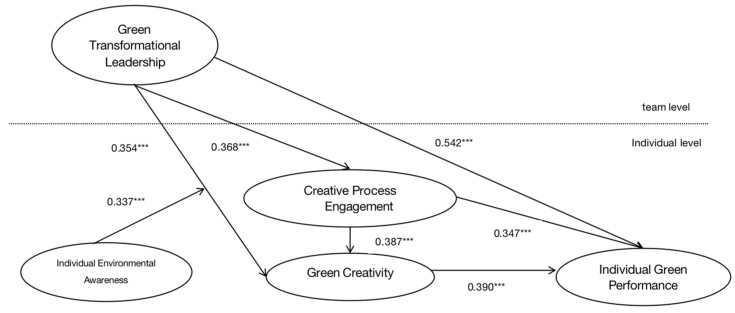
Multilevel structural equation modeling for a robustness test. *** *p* < 0.001.

To assess the reliability of the findings, we performed multilevel structural equation modeling using MPLUS 8.3. Figure 3 provides the overall testing results of the hypotheses. 

The total impact of green transformational leadership on employee green performance was 0.542 (95% CI [0.396, 0.652]), which supports H1. The influence of green transformational leadership on creative process engagement was 0.368 (95% CI [0.236, 0.500]), which supports H2. Furthermore, results showed a positive mediation effect (green transformational leadership → creative process engagement → employee green performance) of 0.127 (95% CI [0.077, 0.178]), which is statistically significant. Therefore, creative process engagement effectively mediates green transformational leadership and individual green performance, supporting H3. The influence of green transformational leadership on green creativity was 0.354 (95% CI [0.222, 0.486]), supporting H4. Results showed a positive mediation effect (green transformational leadership → green creativity → employee green performance) of 0.138 (95% CI [0.077, 0.198]), which is statistically significant. Thus, green creativity mediates green transformational leadership and individual green performance, supporting H5. Considering that there are two mediating paths in this study, we also used the method proposed by Preacher and Hayes (2008) to test the difference in multiple mediating effect sizes [59]. We compared the two mediating paths that can be promoted to increase individual green performance. The results show that the mediating effect of green creativity between green transformational leadership and employee performance (0.138) is slightly greater than that of creative process engagement (0.127). The estimated value of the difference between the two mediation effects (a1b1–a2b2) is 0.011. The 95% confidence interval is [−0.027, 0.031], including 0, indicating that mediating power did not differ significantly between the two paths and the effects are equivalent. The sequential mediation effect of creative process engagement and green creativity between green transformational leadership and individual green performance was 0.055 (95% CI [0.032, 0.079]), supporting H6. Moreover, H7 states that individual environmental awareness moderates the influence of green transformational leadership on employee green creativity. Our research found a significant relationship between the interaction term (green transformational leadership × individual environmental awareness) and employee green creativity (0.337, 95% CI [0.256, 0.418]), supporting H7.

## 5. Discussions

The purpose of our study is to respond to the current shift in research attention from the green performance of the firm to the green performance at the individual level of the firm’s employees, who play a crucial role in planning and implementing innovations. Our study utilizes data collected from Chinese chemical firms to validate the interrelationship between GTL and individual employee green performance through green creativity and creative process engagement as mediating variables and individuals as moderating variables. Thus, our empirical findings provide useful contributions to the green management literature and theory development, while meeting Chinese chemical companies’ practical needs and goals. 

The research results show that GTL positively impacts employees’ individual green performance, confirming the empirical research results of Khan et al. [60]. This approach promotes employees’ confidence and consensus in established values and strengthens competencies and behaviors related to their achievements. Green transformational leaders can use their behaviors to influence employees to engage in creative activities [61], motivating more green behaviors [62]. This study identifies the mechanisms by which chemical company leaders influence the green performance of individual employees in chemical companies. This provides a new perspective from which to test the effectiveness of green transformational leadership and encourages individual-level green performance. Therefore, chemical companies should implement a culture that encourages this. 

In addition, the findings showed a positive effect of CPE and GC in the relationship between GTL and employees’ individual green performance. This finding is consistent with previous studies, which have mostly analyzed the effect of creativity on the psychological dimension and neglected the process of creative engagement [63]. Therefore, this study emphasized the creative process and used the mediating variable of CPE. Although psychological factors are necessary for creative behavior, we should pay more attention to the nature of creative thinking and explore its origins more deeply [50]. Meanwhile, the present paper discusses ways to cultivate employees’ green creativity at both the organizational and individual levels. It broadens the research into how employee creativity can enhance green performance, thus adding information to the theoretical understanding of sustainability and green organizational innovation. By adopting a new perspective, and based on empirical evidence, we conclude that green transformational leadership promotes individual employees’ green performance through a chain-mediated effect on employee creative process engagement and green creativity. 

This study’s moderating variable was individual environmental awareness, which significantly altered the association between green transformational leadership and green creativity. The present findings show that employees who are thoroughly aware of ecological and environmental issues are more inclined to take environmental actions [16]. This finding is consistent with the findings of Mansoor et al. [38]. Numerous studies have demonstrated the beneficial effects of transformational leadership on employee creativity and that this style of leadership fosters employee creativity by increasing employee involvement in environmental protection through increased environmental awareness [64]. This result is interesting and logical, because employees with high environmental awareness can spontaneously participate in environmental activities, and their awareness and commitment to environmental sustainability and sustainable development influence their behavior and decision making, increasing their creativity. In exploring the relationship between green transformational leadership and employees’ green creativity, this study found that IEA significantly affected the relationship. This result verifies a positive correlation between adopting IEA and enhancing the relationship between green transformational leadership and employees’ green creativity. In addition, when employees’ individual environmental awareness is enhanced, green transformational leadership encourages employees’ green creativity by enhancing environmental awareness. Furthermore, this study validates the importance of individual environmental awareness in Chinese chemical companies. 

### 5.1. Theoretical Contributions 

Firstly, academics are increasingly paying attention to environmental leadership. Furthermore, most existing studies have examined this topic from the perspectives of process, competence, and behavior [47]. This paper combined organizational behavior research with environmental management research to consider green transformational leadership as a leadership style and examine the relationship between organizational goals and leaders. The study of organizational behavior explores organizations’ individual and team behaviors and their impact on organizational performance and goals. By combining the study of organizational behavior with the study of environmental management, it is possible to gain a deep understanding of how the behavior of leaders affects the environmental behavior of employees and the environmental performance of organizations [22]. Thus, we can examine the specific behaviors of green transformational leaders, such as the degree to which leaders pay attention to environmental protection issues, the setting and promotion of environmental protection goals, the degree of employee participation in environmental protection decision-making, etc. At the same time, it is also possible to study the relationship between the organization’s environmental goals and leaders’ behaviors and how leaders affect employees’ environmental awareness and behaviors [65]. 

By combining the studies of organizational behavior and environmental management, research can guide leaders and organizations to drive environmentally friendly behavior and sustainable development [44]. This research can help organizations understand how to cultivate green transformational leadership and inspire employees’ environmental awareness and actions through leadership behaviors. This will help organizations achieve environmental sustainability and higher levels of performance [66]. The impact of green transformational leadership and its mode of operation on employee psychology and behavior in China is also further examined in this essay. This study adds to the knowledge of green transformational leadership’s influencing factors. Through this study, we can further understand the influencing factors of green transformational leadership in the Chinese context and provide guidance and suggestions for organizations to cultivate and develop green transformational leadership. This examination helps drive Chinese organizations in a greener, more sustainable direction and enhances employee participation and environmental protection awareness [48]. 

### 5.2. Practical Contributions 

This study contributes to the greening of organizations by proposing the following management recommendations.

Firstly, considering the critical role of green transformational leaders in achieving excellence in green innovation, chemical companies should actively promote this culture and focus on developing green transformational leaders. Particularly in addressing environmental challenges, companies need to actively appoint transformational leaders who are passionate about environmental issues to transform potential crises into opportunities for innovation and organizational development. This study provides new perspectives and practical advice for chemical industry managers to encourage them to apply green transformational leadership as a new leadership style.

Secondly, green creativity acted as a mediator to positively influence each participant’s performance. Green creativity is considered an important factor affecting employee performance [42], and innovation and green performance are closely related to creativity, especially in industries such as chemicals. Enterprises should pay attention to cultivating employees’ green creativity within the company’s organization, and leaders can develop targeted training programs for employees based on current environmental policies and carry out a series of green culture development projects. By fostering a positive green innovation culture, the overall green performance of the organization can be improved [47].

Finally, chemical company leaders should consider individual environmental awareness as a regulatory variable. To increase the environmental awareness of employees, leaders should organize green team-building activities to develop a sense of responsibility and mission towards environmental issues and concern for the community. This approach helps shape employees’ ideas and perceptions, facilitating the organization’s green initiatives. As a developing country, China has more resource-based industries than foreign companies, and its activities generate serious pollution. Chinese chemical companies must choose between destroying the environment or seeking economic benefits through green initiatives, and as the economy globalizes and international competition intensifies, the demand for leadership is increasing. Therefore, the Chinese government should support and encourage chemical companies to advocate and establish a corporate culture of green transformational leadership, create an atmosphere and environment of green transformational leadership, and improve leaders’ green transformational leadership through policy support, education and training, green innovation incentives, and the promotion of information sharing and cooperation, etc. This study is of great practical significance for chemical companies.

### 5.3. Limitations and Recommendations

From a comprehensive standpoint, this paper still has flaws and restrictions, which could affect the representativeness of its results. Firstly, future studies could verify the research findings by broadening the research scope and increasing the industry types and samples. 

Secondly, this study sampled 98 team leaders and 525 employees from five chemical companies in Shandong Province, China. This narrow sample increases the internal validity of the research while lessening the impact of geographical and industrial factors. The potential differences in reactions to the leadership behaviors of green transformational leaders among workers in different areas and sectors limit the study’s external validity. By extending the study’s scope or including workers from different industries as sample interviewees, future studies can increase the study’s external effectiveness. 

Thirdly, because this study focused on a single culture, additional research examining other cultures could enhance its applicability and generalizability. Furthermore, we only surveyed chemical companies in China, and given that Chinese culture follows a social hierarchy, employees may be more affected by their leaders and more inclined to consider them as role models due to inequalities in social rank, power, and authority. In addition, people in China tend to be more impacted by the atmosphere at work because of the country’s collectivist culture; consequently, future research should examine potential cross-cultural differences. 

Fourthly, this paper introduced the moderating variable of individual environmental awareness to examine the impact of green transformational leadership on green creativity. However, we did not test whether there is a moderating effect of individual environmental awareness among other variable relationships. The impact of green transformational leadership on individual green performance may have other moderating variables that affect and regulate the effective transmission of green transformational leadership behaviors. Therefore, future research should identify these moderating variables. Moreover, hypothesizing about the nature of these variables’ moderating roles could enrich the research model.

## 6. Conclusions

This study examines the mechanisms by which green transformational leadership affects individual green performance from different perspectives (i.e., from two different levels: individual and organizational). Specifically, we focused on the chain-mediated effects of employees’ green creativity and creative process engagement, while considering the moderating role of individual environmental awareness. This study provides new insights regarding how green leadership affects individual green performance. The results provide a powerful template for subsequent, related studies and validate the mechanism’s generalizability. In the current global context, the green development of enterprises must be a long-term research topic; however, enterprises must think about how to enhance their employees’ green performance to improve the overall sustainability of their operations. Meanwhile, this study synthesizes a variety of green-related variables to provide valuable practical experience and insights for innovative practices and green management in chemical companies. 

## Figures and Tables

**Table 1:** Employees’ and team leaders’ demographic characteristics.

Employees’ demographic characteristics			
Variables	Dimension	N	Percentage (%)
Gender	Male	344	65.5
	Female	181	34.5
Education Level	High school degree or below	84	16.0
	College degree	162	30.9
	Bachelor’s degree	241	45.9
	Master’s degree or above	38	7.2
Work experience	1–3	169	32.1
	4–6	103	19.5
	7–10	253	48.4
Age range	20–29	148	28.1
	30–39	147	28.0
	40–49	144	27.5
	50 or above	86	16.4
**Team leaders’ demographic characteristics**			
Variables	Dimension	N	Percentage (%)
Gender	Male	65	66.3
	Female	33	33.7
Education Level	College degree	18	18.4
	Bachelor’s degree	45	45.9
	Master’s degree or above	35	35.7
Work experience	8–10	3	3.0
	11–13	21	21.5
	14–16	19	19.3
	17–19	23	23.5
	20 or above	32	32.7
Age range	30–39	14	14.2
	40–49	61	62.3
	50 or above	23	23.5

**Table 2:** Reliability and validity of scales.

Variable	Items	Cr-Alpha	Factor Loading	CR	AVE
Creative Process Engagement	11	0.937	0.721–0.832	0.937	0.576
Individual Environmental Awareness	4	0.889	0.790–0.874	0.892	0.673
Green Creativity	6	0.899	0.689–0.834	0.901	0.602
Individual Green Performance	10	0.922	0.686–0.786	0.923	0.547
Green Transformational Leadership	6	0.890	0.702–0.812	0.892	0.579

GTL = Green Transformational Leadership, CPE = Creative Process Engagement, IEA = Individual Environmental awareness, GC = Green Creativity. IGP = Individual Green Performance.

**Table 3:** Means, Standard Deviations, and Correlations of Variables Studies.

	Mean	SD	1	2	3	4	5	6	7	8	9	10
Team Leaders												
Gender	0.337	0.475										
2. Age	45.122	5.681	0.095									
3. Bachelor’s degree	0.459	0.501	−0.050	0.172								
4. Master’s degree or above	0.357	0.482	−0.216 *	−0.129	−0.687 ***							
5. Work Experience	17.378	4.585	0.154	0.637 ***	0.103	−0.113						
6. Team size	7.214	1.737	0.099	0.007	0.135	−0.129	0.009					
7. Green Transformational Leadership	3.541	0.618	0.015	0.065	−	−	0.010	0.086	0.761			
Team Members												
Gender	0.345	0.476										
2. Age	37.406	10.214	0.031									
3. College degree	0.309	0.462	−0.025	−0.073								
4. Bachelor’s degree	0.459	0.499	0.007	−0.112 *	−0.615 ***							
5. Master’s degree or above	0.072	0.259	0.014	−0.092 *	−0.187 ***	−0.257 ***						
6. Work Experience	7.438	6.069	−0.053	0.692 ***	−0.013	−0.136 **	−0.066					
7. Creative Process Engagement	3.425	0.775	0.009	−0.005	0.054	−0.064	−0.022	−0.034	0.759			
8. Individual Environmental awareness	3.767	0.916	−0.066	0.006	−0.003	−0.051	0.057	0.046	0.065	0.820		
9. Green Creativity	3.727	0.888	−0.013	−0.005	0.038	−0.108 *	0.028	0.002	0.423 ***	0.317 ***	0.776	
10. Individual Green Performance	3.501	0.843	0.033	−0.032	0.042	−0.088 *	0.069	−0.054	0.497 ***	0.124 **	0.532 ***	0.739

* *p* < 0.05, ** *p* < 0.01, *** *p* < 0.001. Nlever1 = 98 (team Leaders); Nlever2 = 525 (team member). Employees’ gender, employees’ age, education level, team leaders’ gender, team leaders’ age, team leaders’ education level, and team size were dummy processed. Employees gender (male = 0, female = 1); employees education level (high school degree = 0, 0, 0, 0; college degree = 0, 1, 0, 0; bachelor’s degree = 0, 0, 0, 1; master’s degree or above = 0, 0, 0, 1); team leader gender (male = 0, female = 1); team leader education levels (college degree = 0, 0, 0; bachelor’s degree = 0, 1, 0; master’s degree or above degree = 0, 0, 1). The square root of AVE is on the diagonal.

**Table 4:** Results of hypotheses testing.

	Null Model 1	Model 1	Model 2	Null Model 2	Model 3	Model 4	Model 5	Model 6	Null Model 3	Model 7	Model 8	Model 9	Model 10	Model 11
Variables	Creative Process Engagement			Green Creativity					Individual Green Performance					
Intercept		3.446 ***	3.448 ***		3.771 ***	3.772 ***	3.771 ***	3.752 ***		3.538 ***	3.539 ***	3.528 ***	3.519 ***	3.516 ***
Level 1														
Employee gender		−0.023	−0.013		−0.004	0.008	0.028	0.019		0.026	0.046	0.047	0.035	0.040
Employee age		0.000	−0.001		−0.002	−0.003	−0.002	−0.001		0.002	0.001	0.001	0.003	0.002
College degree		−0.034	−0.040		−0.132	−0.140	−0.123	−0.116		−0.046	−0.052	−0.035	0.016	0.012
Bachelor’s degree		−0.011	−0.001		−0.131	−0.119	−0.105	−0.097		−0.075	−0.056	−0.052	0.008	−0.005
Master’s degree or above		0.040	0.040		−0.047	−0.039	−0.052	−0.054		0.022	0.050	0.032	0.059	0.044
Work Experience		0.001	0.002		0.003	0.005	0.002	0.002		−0.004	−0.002	−0.002	−0.004	−0.004
Creative Process Engagement												0.504 ***		0.344 ***
Green Creativity													0.511 ***	0.390 ***
Individual Environmental awareness							0.237 ***	0.256 ***						
Level 2														
Leader gender		−0.072	−0.075		−0.052	−0.059	−0.088	−0.084		−0.001	−0.005	0.033	0.025	0.044
Leader age		0.006	0.003		−0.007	−0.012	−0.012	−0.014		0.014	0.009	0.008	0.015	0.013
Bachelor’s degree		−0.103	−0.166		−0.202	−0.293	−0.257	−0.319 *		−0.244	−0.328 *	−0.245	−0.181	−0.159
Master’s degree or above		−0.301	−0.287		−0.374	−0.355 *	−0.285	−0.266		−0.198	−0.173	−0.027	0.009	0.066
Work Experience		−0.016	−0.013		0.001	0.004	0.005	0.011		0.009	0.014	0.020	0.011	0.016
Team size		−0.020	−0.027		−0.009	−0.020	−0.025	−0.040		0.037	0.026	0.040	0.037	0.044
Green Transformational Leadership			0.379 ***			0.549 ***	0.519 ***	0.510 ***			0.540 ***	0.348 ***	0.258 **	0.194 **
Cross-level _GTL × EA								0.347 ***						
R (Sigma squared)	0.332	0.335	0.335	0.393	0.396	0.396	0.374	0.357	0.422	0.427	0.428	0.332	0.306	0.272
U (Tau)	0.276	0.286	0.232	0.400	0.410	0.295	0.258	0.224	0.294	0.291	0.177	0.154	0.172	0.148
Chi-square	522.911 ***	496.706 ***	413.754 ***	628.896 ***	591.699 ***	446.721 ***	421.042 ***	388.494 ***	457.012 ***	420.171 ***	288.192 ***	311.992 ***	363.169 ***	352.283 ***
Deviance	1079.151	1130.408	1115.586	1229.934	1229.934	1205.530	1176.49	1147.276	1190.985	1238.050	1206.188	1089.510	1059.824	1000.257

* *p* < 0.05, ** *p* < 0.01, *** *p* < 0.001.

## Data Availability

Data will be made available upon request.

## Appendix A. Questionnaire

Please circle your desired response as to what extent you agree with that statement. For example, if your response is 4 (Agree), draw a circle around 4.

**Table A1:** Questionnaire.

S. No	Items (Green Transformational Leadership)	Strongly Disagree	Disagree	Neutral	Agree	Strongly Agree
01	My leader provides a clear environmental vision for the followers to follow.	1	2	3	4	5
02	My leader inspires followers with environmental plans.	1	º2	3	4	5
03	My leader gets the employees to work together for the same environmental goals.	1	2	3	4	5
04	My leader encourages human resources to achieve environmental goals.	1	2	3	4	5
05	My leader acts by considering the environmental beliefs of the individuals.	1	2	3	4	5
06	My leader stimulates subordinates to think about green ideas.	1	2	3	4	5
**S. No**	**Items (Creative Process Engagement** **)**	**Strongly Disagree**	**Disagree**	**Neutral**	**Agree**	**Strongly Agree**
1	I spend considerable time trying to understand the nature of the problem.	1	2	3	4	5
2	I think about the problem from multiple perspectives.	1	2	3	4	5
3	I decompose a difficult problem/assignment into parts to obtain greater understanding.		2	3	4	5
4	I consult a wide variety of information.	1	2	3	4	5
5	I search for information from multiple sources (e.g., personal memories, others’ experiences, documentation, Internet, etc.)	1	2	3	4	5
6	I retain much detailed information in my area of expertise for future use.	1	2	3	4	5
7	I consider diverse sources of information in generating new ideas.		2	3	4	5
8	I look for connections with solutions used in diverse areas.	1	2	3	4	5
9	I generate a significant number of alternatives to the same problem before I choose the final solution.	1	2	3	4	5
10	I devise potential solutions that move away from established ways of doing things.	1	2	3	4	5
11	I spend considerable time shifting through information that helps to generate new ideas.	1	2	3	4	5
**S. No**	**Items (Green Creativity)**	**Strongly Disagree**	**Disagree**	**Neutral**	**Agree**	**Strongly Agree**
1	I can come up with new ways to achieve environmental goals.	1	2	3	4	5
2	I can develop new environmental concepts to improve the organization’s environmental performance.	1	2	3	4	5
3	I can promote and advocate new green ideas to others.	1	2	3	4	5
4	I can develop adequate plans to implement new environmental concepts.	1	2	3	4	5
5	I reconsider new green ideas.	1	2	3	4	5
6	I will find creative solutions to environmental problems.	1	2	3	4	5
**S. No**	**Items (Individual Environmental awareness)**	**Strongly Disagree**	**Disagree**	**Neutral**	**Agree**	**Strongly Agree**
1	The effects of pollution on public health are worse than we realize.	1	2	3	4	5
2	Over the next several decades, thousands of species will become extinct.	1	2	3	4	5
3	Claims that current pollution levels are changing the earth’s climate are exaggerated (reverse coded).	1	2	3	4	5
4	Environmental protection will provide a better world for me and my children.	1	2	3	4	5
**S. No**	**Items (Individual Green Performance)**	**Strongly Disagree**	**Disagree**	**Neutral**	**Agree**	**Strongly Agree**
1	In his/her work, this employee weighs the consequences of his/her actions before doing something that could affect the environment.	1	2	3	4	5
2	This employee voluntarily carries out environmental actions and initiatives in his/her daily work activities.	1	2	3	4	5
3	This employee suggests to his/her colleagues how to protect the environment more effectively, even when it is not his/her direct responsibility.	1	2	3	4	5
4	This employee spontaneously gives his/her time to help his/her colleagues take the environment into account in everything they do at work.	1	2	3	4	5
5	This employee encourages his/her colleagues to adopt more environmentally conscious behavior.	1	2	3	4	5
6	This employee encourages his/her colleagues to express their ideas and opinions on environmental issues.	1	2	3	4	5
7	This employee actively participates in environmental events organized in and/or by our organization.	1	2	3	4	5
8	This employee stays informed of our organization’s environmental initiatives.	1	2	3	4	5
9	This employee undertakes environmental actions that contribute positively to the image of our organization.	1	2	3	4	5
10	This employee volunteers for projects, endeavors, or events that address environmental issues in our organization.	1	2	3	4	5
